# An Inquiry into the Causes of the Motion of the Blood' &c.

**Published:** 1816-06

**Authors:** 


*An Inquiry into the Causes of the Motion of the Blood, &c.* By James Carson, M. D. &c. 8vo. pp. 250. London, 1815.

The title of the foregoing work; some accounts of its *unintelligible* contents; the unsatisfactory results of our own reading and reflection on the subject, all conspired to prepossess us with gloomy anticipations on our sitting down to its perusal. We were most agreeably disappointed; for we have seldom read a work of a *speculative* nature with which we were more pleased or more interested. We will not conceal one cause of this pleasure; namely, that some coincidence of opinion on many important bearings of the subject discussed, must have existed between the author and ourselves, anterior to the publication of the present work.

In the first Number of the Med. Chir. Journal, while reviewing Dr. Parry's work, we took occasion to dissent from that acute physician on the circulation of the blood. Dr. P. with Haller maintains, that the force of the heart *alone*, by which he means the *arterial* side of the heart, is adequate to the task of carrying on the march of the blood through its entire circulation.

"Hence," says he, "at every systole, or contraction of the left ventricle of the heart, the shock acts at the same instant throughout the entire circle." p. 34.

We there expressed our dissent, but did not stop to give our reasons. The present volume of Dr. Carson's will convince the most sceptical, that Dr. Parry's conclusions on the above point are very erroneous. According to this doctrine, the *auricles* of the heart would be little else than useless *appendages* to that organ. If they merely transmitted the blood they received from the venous system, into the ventricles, why might not the ventricles have received it from that source themselves? We were ever of opinion, that the auricles of the heart exerted by their *dilatation* an active influence on the venous blood, pumping it in fact from that languid system of circulation so opposite to the arterial. We could not, however, account satisfactorily for the manner in which this *forcible dilatation*, or suction was performed. We ascribed it to an inherent power in the auricle, similar to contraction, for which we cannot account. It is this obscure point which Dr. Carson endeavours to clear up ; and here we cannot help observing, however it may offend some of our brother critics, that to us Dr. Carson's language appears as perspicuous, as his ideas are clear and well arranged. There is, indeed, a mathematical precision, as well as a terseness in our author's reasonings and style which are highly creditable to his talents.

It is impossible to analyse such a volume without doing great injustice to the force of the ingenious author's reasonings and experiments, and therefore we can do little more than lay his results before our readers; recommending a perusal of the volume to every one who wishes to form an accurate idea of the blood's circulation. It is true, that the student, as well as the physician, can tell you, like a parrot, the exact rout of the vital current, with every avenue and bye-way through which it passes; but when they have perused Dr. Carson's Essay, they will be astonished to find how little they knew about the matter ; and how many difficulties were passed over without a thought!

The great originality in this Essay, is that part in which the dilatation of the cavities of the heart is accounted for. Dr. C. observes, as is admitted by anatomists, that the substance of the lungs is powerfully elastic; and that from the first inspiration of the infant, the lungs are swelled into a forced, or unnatural state of expansion; that is, that they are distended into dimensions far more ample than those which they would assume, if all force or restraint was withdrawn from them. This forced dilatation is evidently the effect of the weight of the atmosphere. We need not stop to prove this :—the collapsed state of the fœtal lungs, and the collapse consequent on making an opening into the thorax are sufficient. There is, therefore, a constant tendency in the lungs to collapse and retire from the internal surface of the thorax ; an effort at separation measurable by the share of atmospherical pressure which the elastic power of the lungs is, at the instant, capable of supporting: But as this power is by no means equal to the whole weight of the atmosphere, and as no elastic substance intervenes between the lungs and the cavity of the thorax, no actual separation can be produced.

"It is further evident, that all the parts which compose the walls or boundaries of the chest, are pressed, or, in common language, drawn towards the spaces from which the lungs have a tendency to retire, with a force which is commensurate with the elastic power of these organs, or with the share of atmospheric weight which that power is at the instant capable of supporting. The bony structure of the thorax is unyielding; the diaphragm is forced to assume a tense convexity towards the centre of pressure.

"In what condition is the heart in these circumstances? It must necessarily be considered as constituting a part of the walls or boundaries of the chest; for there is a free communication between its internal cavities and the parts without the thorax, as well as the substance of the lungs, particularly with the mass of fluids through the bores of the large venous trunks. Between the external or convex surface of the heart and the surface of the lungs, membranes alone intervene. The divided laminæ of the mediastinum are thin, pliant, easily dilatable, and evidently incapable of supporting, either on the upper or lower surface, any share of preponderating pressure. The pericardium, indeed, which surrounds the heart, and which is every where in contact with it, is a strong, firm, inelastic bag, and cannot be expanded beyond certain limits, without such a powerful force as would at the same time injure its structure; but of itself it will not resist any pressure that may rest upon its external surface. It is fitted to resist with ease the balance of unequal pressure, which may be laid upon its internal or concave surface by the collapsing effort of the lungs: but, before it can be brought to support any share of this balance, it must be dilated by its contents to the utmost extent. Until the pericardium has been extended to the extreme limits of its expansibility, the unequal balance which the boundaries of the chest have every where to support, must be resisted at this part by the walls of the heart. A force equal to the weight which the elasticity of the lungs, in their ordinary condition during life, is capable of supporting, must constantly tend to dilate the heart to the utmost limits of the pericardium, by ponderating against the internal surfaces of its chambers; and until these chambers are expanded in such a degree as would swell the general dimensions of the heart to an equality with the capacity of the pericardium, this force must all be sustained by the walls of the heart itself. As the pericardium cannot be distended further, through any other means than the expansion of the chambers of the heart, and as a full dilatation of at least the greater part of those chambers would be required to give to the heart dimensions commensurate with the utmost limits of the cavity which the pericardium is capable of inclosing; the successive dilation of the different chambers of the heart after contraction is fully secured by an adequate power.

"By removing, therefore, in consequence of their elasticity, a part of the pressure of the atmosphere from the convex surface of the heart, and by that means causing the ordinary pressure to ponderate unequally against the convex surfaces of the chambers, the lungs become the certain and powerful antagonists of the muscles of the heart. The contractile force of the heart is necessarily more powerful than the antagonist action derived from the elasticity of the lungs, but the former is transient and interrupted, while the latter is equal and permanent; during the intervals between the exertion of the muscular energy, it becomes predominant; and, co-operating with the natural tendency of the structure, restores the chambers of the heart to the state from which they had been forced, and at which the superior strength of the contractile power begins again to be exerted." 118.

This is, perhaps, the most abstruse part of the work; but we think we can clearly comprehend the meaning, though we do not implicitly subscribe to the theory. We believe, however, the *effect* which Dr. C. thinks is produced in the above manner, but we are not quite satisfied with the cause, or modus operandi.

That the ventricular or arterial force of the heart and arteries is not sufficient to carry the blood round the whole course of the venous circulation is ably proved by Dr. Carson. Among other arguments, we may notice the following:—If a ligature be made on a single vein, or on a whole limb, and that limb be kept at rest, the blood will forsake that portion of the vein or veins *above* the ligature as fast as though no ligature was applied.—Where can the *vis a tergo* act here?—Muscular propulsion too, although it be capable of accelerating the circulation, has not so much power in this way as is generally supposed. Indeed, it is not likely, that nature should leave so important a concern as the venous circulation at the mercy of the action of voluntary muscles. But if the latter, as in exercise, can quicken the circulation, how is it, that the increased impetus of the blood continues *after* the muscles are at rest, as we may easily satisfy ourselves is the case. This must either proceed from the *vis a tergo*, or from auricular suction, or both. We have shewn that the *vis a tergo* cannot be equal to the whole march of the circulation; and consequently, the suction influence of the heart must co-operate. The veins themselves are not well adapted for carrying forward the blood to the heart, independently of an attraction from that viscus. The veins are supposed to contain at least double as much blood as flows in the arteries; and as the calibre of the *branches*, collectively, infinitely exceeds that of the venæ cavæ, the vital current in approaching the heart is constantly passing into a narrower channel. To this may be added, the collision of currents flowing from frequent anastomoses, and meeting at considerable angles; by which, the momentum of each is more or less expended. From these, and various other phænomena which might be stated, we may safely conclude, that the *vis a tergo* contributes a part only in the venous circulation.

Dr. Carson ingeniously supposes, that, as an *elastic* substance is, in the heart, every where mingled with the muscular, its elasticity may be constituted in such a manner as to resist the contractile effort of the muscle. The natural position of the elastic substance in every fibre must be the same with that of the muscular; and consequently, any alteration in the elastic fibre, produced by the muscular portion acting according to its natural direction, must be to contract or expand the elastic, according to its natural direction. Therefore the elasticity, if it is an antagonist power to the muscular, must tend to restore the fibre to the same position relatively that it had before it was contracted. There arises, therefore, from the circular direction of the fibres, (as ascertained by Stewart) and the arched form of its walls, a very remarkable property—that of dilating or expanding its cavities by the simple relaxation of its fibres. The force, indeed, with which the heart is capable of expanding its cavities, we cannot exactly ascertain; but it must be increased by the spirit and energy of life, to a degree of which we can form no adequate conception, by an examination of the dead fibre.

The force of gravity on the venous circulation is thus described by Dr. Carson.

" The blood in the vena cava and its divisions may be supposed to be placed, with respect to that in its smaller ramifications, between which, anastomoses generally prevail, and which, on that account, may be deemed a single vessel, in such a manner as to constitute an opposing or balancing column. As the fluid is in both of the same specific gravity, the height of it in the one column will, unquestionably, be regulated by its height in the other. Let it be supposed then, that the system of the vena cava has been filled with blood, and that this blood has subsided in all its parts to an adjusted equilibrium, and let this system, in the state supposed, be subjected to the action of the heart and arteries, in the same manner that has been described. By the stroke of the heart, a quantity of fluid is withdrawn from one end of the column; and by the synchronous vibrations of the arteries, an equal quantity is added to the other. While the adjusted level of the fluid is diminished on one side by the abstraction of a part, it is heightened on the other by the accession of fresh matter. A double derangement is the effect. By these actions, a motion must be excited in the fluid generally, to restore the equilibrium between the different parts of it. But the causes are perpetually renewed; therefore, a perpetually repeated generation of motion must be produced through the different parts of the venous system by gravity, and this motion must be from the ends of the veins to the trunks.

"It is immaterial from what part of the column, in relation to height, the portion of fluid be abstracted, or to what part the addition be made; the derangement of equi-ponderance in the fluid generally, will evidently, in every case, be the same.

"It appears then, that in this manner, gravity augments the impulse which the venous blood sustains from the heart and arteries; and, in all probability, produces a great proportion of that which has been denominated the positive motion of the blood in the veins, and which is indicated by the swelling observable in the part of the vein beyond the ligature.

"The force communicated to the venous blood by the heart and arteries, assisted by gravity, and favoured by the natural structure of the vessels, may be supposed to be sufficient to preserve any part of the venous system from a permanent state of collapse; and thus, as the actions of the heart are renewed, to place a fresh supply of blood within the space of its influence. The blood thus solicited and impelled, continues to flow, in an uniform continued stream, from the ends of the veins to the heart." 140.

The smooth and equable current of venous blood, apparently sustaining no impulse from any other substance, nor producing the slightest agitation in the flexible tubes through which it flows during all the vicissitudes of its course—subject to rapid changes of direction—perpetually varying its elevation, and formed by apparently opposing streams, is a matter that is calculated to strike the mind with astonishment!

The motion of a fluid so rapid, but at the same time so imperceptible and quiet, as that of the blood in the veins, seems to be deducible only from the principles stated—namely, a vis a tergo, an auricular suction, and an atmospheric pressure, or influence of gravitation. If this pressure be, to a certain degree, removed from any part of this body of fluid, the particles, on one side of which the pressure has been removed, will yield to the greater influence on the other side, and advance, and so on till a motion is produced in the whole.

We shall here present our readers with a brief recapitulation of the causes by which the circulation of the blood is supposed to be effected, combined with a summary explication of the manner, and so far as that can be ascertained, the proportion in which each contributes its share.

"By every contraction of the ventricles, a quantity of blood is projected forcibly into the great arteries. As the coats of the arteries are dilatable, these vessels give way to the impetuosity of the propelled blood; and, to a certain distance from the heart, become of a more enlarged calibre. In consequence of the stimuli derived from the blood newly admitted into the arterial cavity, and (if so bold a metaphor may be used) from the pain of distention, the irritability of the muscular fibres is excited; the contractile effort is roused; and, co-operating with the elastic, restores the artery, by a rapid movement, to its former dimensions. As the blood is an incompressible fluid, a quantity equal to that projected from the heart, must now be displaced from that portion of the arterial system which had been dilated by the immediate impulse of the ventricles. The valves, which are stationed at the roots of the great arteries, and which are securely closed, during the contraction of the first portion of the arterial system, by the retrograde movement of the blood nearest the heart, and more effectually by the suction occasioned by the synchronous dilatation of the ventricles, completely prevent the return of any fluid from the arteries into the heart; while the more ample calibre possessed by the aggregate of the vessels beyond the contracting portion, and distensible condition of their relaxed fibres at the moment, favour its advance into a more distant portion. This portion, now distended and stimulated by the same causes by which the first had been excited, recoils vividly upon its contents; and by the rapid resumption of its former capacity, expels from its cavity a quantity of blood equal to the addition which it had received during its dilatation. The blood, displaced by this movement from what may be termed the second portion of the arterial system, is directed into a more advanced part of it, by the different state of the coats of the vessels and the difference between the aggregate of their calibre, before and behind the contracting portion; because, in consequence of the rapidity of vibratory undulation, the coats of the vessels between the contracting portion and the heart are still rigid, while those beyond it are relaxed and easily dilatable; and because the outlets on this side are greater, as the aggregate of the bores of the vessels increases with the distances from the heart. The same series of actions is repeated to the ends of the arterial system. A quantity of blood, therefore, equal to that which had been projected from the heart into the arteries, is propelled from the ends of the arteries into the veins in equal times. The power of the heart may thus be said to be transmitted undiminished to the end of the arterial system.

"No data were discoverable from which the momentum of the blood, discharged from the ends of the arteries, or the quantity of motion it would generate in the venous blood, could be estimated. But from the mass of fluid that was to be put in motion; from the velocity with which it was known to flow; from the form and position of the vessels containing it; from the resistance opposed by friction, tenacity, and various other causes; and from the phænomena attending the venous circulation; it was concluded, with the fullest confidence, that the blood could not be circulated through the veins by the impulse it received from the arteries alone.

"In searching for the causes by which the chambers of the heart were dilated after contraction, it was ascertained, that this condition of the organ was in part to be ascribed to the form and position of its fibres, in consequence of which simple relaxation was accompanied by a certain degree of dilatation; but particularly to the supporting of a part of the atmospherical pressure that would have rested upon the convex surfaces of the heart or its envelope, by the resilient or collapsing effort of the lungs. It was urged, that the abstraction of a part of the ordinary pressure of the atmosphere, from the convex or external surface of the heart, or from the convex surface of the pericardium, was perpetual, and was therefore always ready to co-operate with the dilating faculty of the heart itself, as that was alternately renewed; and that the conjunction of those powers was fitted, during the intervals of contraction, to dilate the chambers to the utmost extent, or at least to the extent of the capacity of the pericardium.

"In consequence of the dilatation of the ventricles by the causes which have just been stated, the valves at the roots of the arterial trunks, yielding to the greater pressure from without, become securely closed, and the resumption of blood by the heart from the arteries is completely prevented, but the passage of blood from the auricular into the ventricular cavities is not obstructed; the blood, therefore, by which the former chambers were dilated, pursues the less resisted course, and occupies the space left by the dilating ventricles. Any deficiency in the full dilatation of the ventricles, which in the healthy condition of these parts can scarcely occur, will readily be supplied by the projectile force of the contracting auricles. By the dilatation of the auricles, the valves, in the auricular passages, sustaining less resistance on their internal surface, become securely closed; but, at the other openings, those by which they communicate with the venous trunks, no obstacles are interposed. The blood, therefore, in the large venous trunks, is relieved from a part of the ordinary pressure in the direction of the heart; it necessarily takes the course in which it meets with least resistance, and continues to move in that course until the resistance is equalized by the full dilatation of the auricles.

"The heart, therefore, acts at once in a two-fold capacity; by the contraction of the ventricles it propels the blood through the arteries, and by the dilatation of the auricles it pumps it from the veins. It is at the same time a forcing and a suction pump.

"The structure of the veins is not fitted for raising the blood through them to the heart, from parts at a distance from that organ, by suction alone: for these vessels being very thin and pliant, would immediately collapse and become impervious under such an influence. Other agents are required to preserve the permeability of the venous vessels, to give them as it were the property of rigid tubes, and to bring the blood generally which they contain within the sphere of the action of the heart. For this purpose, two causes are supposed to co-operate. The first, to which allusion has already been made in this recapitulation, is derived from the projectile power of the ventricles, transmitted by the vibration of the arteries to the ends of the veins. This is the *vis a tergo*, so famous in the schools of medicine. We were unable to estimate the share of the venous circulation that is to be attributed to this cause; which, however, so far as it extends, is evidently well fitted to co-operate with an abstraction of resistance in front, and to preserve uninterrupted the communication between the blood in the remote part and the heart.

"The other power, which was supposed to assist in preserving the distension of the venous vessels in opposition to the suction influence of the heart, was gravity. By the anastomoses which so generally prevail among the veins, particularly among the smaller ramifications, the system of the vena cava may be considered as a single canal. By its retiform fabric, the communication between the blood in the different branches, by the aggregate of which any portion of this canal is formed, is preserved as ready and free as if the blood had flowed in that portion in a single unpartitioned channel. The position of the vessel is fixed. At the moment in which the equilibrium between the contents of the vessel may be supposed to have been adjusted, a quantity of blood is abstracted from one end of it by the stroke of the heart, while a quantity is added to the other by the synchronous contraction of the ultimate portion of the arteries. The balance between the opposing columns of fluid is disturbed; to restore the equilibrium, deranged as the actions of the heart are renewed, a motion is generated in the blood by gravity from the ends of the veins to their roots.

"In short, the motion of the blood while it flows in the veins, is produced by the force of the heart and arteries urging it behind; by the abstraction of a share of the atmospheric pressure from it in front, in consequence of the resiliency of the lungs, interposing its influence in the intervals between the contractions of the heart; and by gravity, which is rendered available in this case by the projection of the arteries and the diastole of the auricles." p. 151.

In the third part of Dr. Carson's work, and in the Appendix, various objections to the theory are ingeniously combated, particularly that of the *fœtal* circulation, where the lungs are not in play; but our closing limits warn us against much farther prolonging our extracts. Dr. C. also applies his theory of the circulation to the explanation of several phænomena which have hitherto puzzled the medical philosopher. We shall here cite one, namely, the adhesion of parts which have been divided or separated from the body.

"When a wound is made on the body, and the parts brought together, and kept in that state, the plastic nature of the blood that had oozed out of the arteries, drying near the surface, unites the edges of the wound. But the blood oozing out of the arteries below the surface, remains liquid. The veins, in the mean time, have become emptied in consequence of the blood which they contained having taken the less resisted course, and returned to the heart. The blood then, which had flowed from the mouths of the arteries into the interstices between the divided surfaces, being less resisted towards the mouths of the veins than in any other direction, necessarily enters the veins, and continues its course to the heart. Other blood follows, and thus a communication is established between the artery and the opposite vein. The blood forming this slow current, coagulates upon the external surface, and in due time, a vessel is thus formed between an artery on one side, and a vein on the other side of the wound."

The reason why no valves are stationed between the cavæ and right auricle is thus given.

"Synchronous with the contraction of the auricle is the dilatation of the ventricle. Consider the causes by which this last chamber is dilated.—They are not the forces which are supposed to proceed from the contracting auricle, and which singly would unquestionably drive the blood as forcibly back into the cava as forwards into the ventricle; they are derived, in part, from the simple relaxation of fibres, which is necessarily accompanied with an expansile effort; but chiefly from the elevation of a share of that pressure, to which all the objects on the face of this globe are subjected, from the convex surface of the heart. The blood, therefore, pressed upon by the contracting auricle, necessarily takes the less resisted direction, and is all drawn into the ventricle. The difference of resistance which the blood has to encounter in the two passages, serves all the purposes of the obstructing valve, which, under different circumstances, would be required at the root of the vena cava. But admitting even that the valve, after which Anatomists have so long searched with scrutinizing eyes, were to be found, it would not serve the purposes which they have had in prospect. Supposing a valve to be stationed at the opening between the cava and the right auricle, and so constructed as to oppose, in similar circumstances, the passage of a fluid from the auricle into the vein; and suppose that the auricle, which has just been filled from the vein, contracts, what in this case is to shut the valve? There is no diminution of resistance or suction influence, which is necessary for the favourable play of valves, on the side facing the vein; on the contrary, the blood is supposed to advance against it with considerable momentum; and the pressure which is made upon the valve by the contracting auricle, is equal on both surfaces. The valve, therefore, would as likely be continued open as closed."

We shall here close our analysis of Dr. Carson's volume: it has afforded us both amusement and instruction, and we recommend it to the perusal of every person who wishes to possess an accurate idea of this most interesting subject. It is highly creditable to the talents of its author, and sincerely do we hope to see something still more useful to the profession from the same able pen.

---

### PART III.

### SELECTIONS.

### FRENCH DICTIONARY OF MEDICAL SCIENCES.

